# Uncertainty and Fertility in Ukraine on the Eve of Russia’s Full-Scale Invasion: The Impact of Armed Conflict and Economic Crisis

**DOI:** 10.1007/s10680-024-09713-7

**Published:** 2024-09-10

**Authors:** Brienna Perelli-Harris, Theodore Gerber, Yuliya Hilevych

**Affiliations:** 1 University of Southampton, Southampton, UK; 2 University of Wisconsin, Madison, USA; 3 University of Groningen, Groningen, The Netherlands

**Keywords:** Fertility, Childbearing, Uncertainty, Ukraine, Armed conflict

## Abstract

While uncertainty has been a key explanation for very low fertility throughout Europe, few studies have analysed how macro-level uncertainty trickles down to shape how people think about having children. Most research focuses on economic uncertainty, not political or social uncertainty. We address these gaps with qualitative data from Ukraine, which has experienced extreme political uncertainty and, for the past decade, armed conflict. Ukraine also had exceptionally low fertility, with an estimated total fertility rate of 1.17 in 2021. In July 2021, we conducted 16 online focus groups on topics related to childbearing with informants living in urban and rural areas in Eastern Ukraine, including areas of Donetsk province that were outside Ukrainian government control. Half the groups consisted of persons displaced by the 2014 Donbas war. The discussions revealed distinct patterns whereby experiences of displacement, the simmering armed conflict, and economic problems combined to produce and intensify uncertainties that discouraged couples from having more than one child. Some blamed the government or delved into conspiracy theories. Armed conflict generates its own forms of uncertainty that interact with persistent economic challenges, dampening fertility.

## Introduction

Uncertainty has been a pervasive explanation for the postponement of childbearing and the widely observed declines to low rates of fertility (Kohler et al., 2002; Mills & Blossfeld, 2005; Vignoli et al., 2020). Most studies in the European context construe uncertainty primarily in economic terms, either with respect to individual-level employment or financial concerns (Alderotti et al., 2021), or macro-level recession and economic crisis (Goldstein et al., 2013; Matysiak et al., 2021; Sobotka et al., 2011). Yet uncertainty can arise from other sources, such as political unrest, military conflict, disease, or pandemics (Trinitapoli, 2023; Trinitapoli & Yeatman, 2011). These macro-level sources can trickle down into individual decision-making processes, affecting how spouses relate to each other, plan for the future, and feel secure in their material and psychological outlook. In contrast to the extensive literature on economic uncertainty and low fertility, the potential impact of other forms of uncertainty, especially armed conflict and displacement, has not been fully elucidated.

Here, we examine how multiple forms of uncertainty influenced childbearing decisions in eastern regions of Ukraine in July 2021, 6 months before Russia’s full-scale invasion on February 24, 2022. These regions had been wracked by political and economic instability after the breakup of the Soviet Union. In early 2014, Russia forcibly annexed Crimea and backed an armed uprising in two eastern provinces (Donetsk and Luhansk), producing 1.5 million internally displaced persons (IDPs) (IOM, 2021). The ensuing stalemate left parts of the “Donbas”, where three million people had resided, as “non-government controlled areas” (NGCAs). Intermittent shelling and violence made it difficult and dangerous to travel across the border, especially during the COVID lockdowns of 2020–2021.

Over the past few decades, demographic estimates for Ukraine have been scarce and imprecise. Ukraine’s last full census was in 2001, and few nationally representative surveys have been conducted since.^1^ This lack of reliable statistical information puts a premium on alternative approaches to understand fertility dynamics. In this study, we conducted 16 online focus groups (by Zoom) in June and July 2021 with informants residing in four locations within eastern Ukraine, including four in the NGCA. While not meant to be representative of the general population, focus group research allowed us to explore social norms and attitudes in greater depth, lending insights into how different dimensions of uncertainty relate subjectively to perceptions about low fertility, ideal family size, and childbearing intentions.

We asked participants about attitudes towards family formation, perceptions of population decline, and low fertility at local and national levels. We also probed to understand how civil unrest, armed combat, and displacement produced life uncertainty. We addressed two overarching research questions: What were the primary reasons that fertility declined in Ukraine, according to the perceptions of Ukrainians? Specifically, did uncertainty or instability factor in their perceptions, and if so, what were the main dimensions of uncertainty relevant for decisions about childbearing?

Our study contributes to the fertility literature in the following ways. First, we bring a qualitative approach to better understand how uncertainty affects fertility. Quantitative methods cannot explore the cognitive and social processes through which economic and political conditions shape considerations and concerns about childbearing. While most researchers speculate about an empirical “black box”, our study provides observation about how these processes operate. Focus groups can elicit shared social perspectives while revealing disparate opinions. Conducting them online allowed us to conduct research in locations nearly impossible to reach otherwise. Second, our focus on armed conflict and displacement, in conjunction with economic crisis, provides a unique perspective on uncertainty. Although many low-fertility European societies have experienced economic recession, Ukraine is the only very low-fertility country in the world to experience war. At the time of our study in 2021, Ukraine had one of the world’s lowest fertility rates, with a Total Fertility Rate of 1.17 (State Statistics Service of Ukraine, 2022). Our study illuminates how these low fertility rates emerged by exploring different dimensions of fertility patterns, including postponement and parity progression.

Barely six months after our focus groups, Russia launched a full-scale invasion of Ukraine, wreaking immense damage to residential areas and civilian infrastructure. The cities and villages where our research took place have become sites of mass atrocities (Human Rights Watch, 2023). Although we do not know the fate of our participants, they may have fled their destroyed homes, live in occupied territory, or become the victims of war crimes. The stories of the IDPs in our research have become much more relevant as the experience of displacement and conflict has intensified. Current estimates indicate that at least one quarter of Ukraine’s population (10 million) have been displaced from their homes since the start of the war (IOM, 2023). As of this writing, it is impossible to predict how the war will end. Whatever the outcome of Russia’s assault, the challenges of reconstruction will be monumental, and concerns about low fertility and attitudes towards childbearing will be of central importance. Although our July 2021 focus groups do not reflect the enormity of the collective trauma felt by the Ukrainian population since February 24, 2022, they do shed light on how this population might approach recovery. We conclude by outlining how insights from our study may be pertinent to Ukraine’s future fertility and population dynamics in the war’s aftermath.

## Background

### Uncertainty and Fertility

Demographers have recently emphasized uncertainty as a fundamental factor in childbearing decisions (Trinitapoli, 2023; Vignoli et al., 2020). As it is impossible to predict the future, uncertainty is a constant feature of the social world that shapes individual’s lives (Trinitapoli, 2023). Uncertainty also influences whether individuals pursue marriage and childbearing now, or postpone until later. Thus, uncertainty may affect either the quantum—the number of children people desire and subsequently have—or the tempo of fertility—the actual timing of births (Trinitapoli & Yeatman, 2011). Certain contexts, such as Africa, have been identified as having high levels of instability that result in unpredictable life course biographies (Trinitapoli & Yeatman, 2011, 2018; Johnson-Hanks, 2004; Agadjanian, 2005).

In European contexts, demographers have attributed declining fertility to economic uncertainty (Vignoli et al., 2020), produced by globalization (Mills & Blossfeld, 2005) exacerbated by economic recession (Sobotka et al., 2011), and operating at both macro- and micro-levels (Kreyenfeld et al., 2012). Employment uncertainty is a particularly common explanation for the postponement and curtailment of childbearing in Eastern and Southern Europe (Alderotti et al., 2021; Gatta et al., 2022; Lebano & Jamieson, 2020). Macro-analyses of economic uncertainty that examine unemployment rates, consumer confidence, and economic policy can explain some, but not all, of the decline in European fertility after the Great Recession of 2008 (Matysiak et al., 2021; Comolli & Vignoli, 2021; Goldstein et al., 2013). On the micro-level, job instability and precarious employment associated with the recent economic crises have also been associated with having fewer children (Alderotti et al., 2021; van Wijk et al., 2021). Economic uncertainty accompanied by low social trust has had a particularly detrimental impact on fertility in settings experiencing recession (Aassve et al., 2020). Subjective economic uncertainties, e.g. worries about future finances, may also influence childbearing (Gatta et al., 2022). A pessimistic future outlook or dismal “narrative of the future” may negatively shape fertility intentions and behaviours (Vignoli et al., 2020).

Nonetheless, not all studies have found a negative association between economic uncertainty and fertility, and some have even found a positive association, for example, in post-Soviet Russia (Kohler & Kohler, 2002). Having children could be a way to reduce uncertainty (Friedman et al., 1994) or provide meaning in one’s life (Edin & Kefalas, 2011). These contradictory findings call for examining the processes by which poor economic conditions and political instability trickle down to influence childbearing decisions.

### Armed Conflict and Fertility

Investigations of the relationship between armed conflict and fertility have found mixed results and examined only high fertility societies. During and shortly after World War II fertility increased in Western contexts (van Bavel & Reher, 2013). In low-income societies, war and forced displacement usually increase fertility, for example, in Colombia (Castro Torres & Urdinola, 2019) Azerbaijan (Torrisi, 2020), Angola (Agadjanian & Prata, 2001) and Burundi (Verwimp et al., 2020). Studies on the fertility of displaced populations also report evidence for fertility recuperation (e.g. Castro Torres & Urdinola, 2019; Torrisi, 2020; Verwimp & Van Bavel, 2005). This increase in fertility may occur for several reasons. First, conflict may interrupt reproductive services, restricting access to contraception or abortion clinics, thus leading to more unintended childbearing. War heightens women’s exposure to unprotected sex and rape, particularly among refugees in camps (Verwimp et al., 2020). High mortality may also induce a response as refugees attempt to ensure a greater number of surviving children for the future (Verwimp & Van Bavel, 2005). Risk insurance and replacement effects may be pronounced for those who lost a child during the conflict (Torrisi, 2020).

However, forced displacement can also result in fertility postponement or decline, as occurred in Karelia, Finland, in the 1940s (Saarela & Wilson, 2022). Forced displacement is disruptive for several reasons (Agadjanian, 2018). Displaced persons often face continued mobility and unstable housing conditions for years (Verwimp et al., 2020; Zavisca et al., 2023). Couples can become separated, with one partner leaving to seek safety or join family members, while the other stays behind or is conscripted into the military. Finally, trauma associated with exposure to violence may create a lasting sense of precarity that can deter couples from having children. Combined with financial insecurity, the instability caused by forced displacement can result in prolonged uncertainty and a pessimistic outlook not conducive to childbearing.

### The Ukrainian Low Fertility Context

Ukraine’s total fertility rate (TFR) began falling in the 1950s (Romaniuk & Gladun, 2015), with some regions in eastern Ukraine declining to below the replacement level already in the 1960s (Hilevych, 2016). However, in the 1990s fertility fell dramatically to very low levels (below 1.3), and in 2001 Ukraine experienced the world’s lowest TFR (1.1) (Perelli-Harris, 2005, 2008). The 2000s saw some recuperation, with the TFR reaching 1.53 in 2012 (State Statistics Service of Ukraine, 2022; Haub, 2014) due to the end of tempo distortions from first birth postponement and increased quantum of childbearing, including second and higher-parity births (Goldstein et al., 2009). Economic recovery during the 2000s apparently ended very low fertility by enabling more couples to achieve their desired fertility. However, the national TFR started falling again after 2012, reaching 1.17 in 2021 (State Statistics Service of Ukraine, 2022).

In Ukraine, as in other parts of Europe, some of the steepest fertility decline reflected the postponement of first births due to mass unemployment, falling incomes, and financial precarity (Kohler et al., 2002). However, the mean age at first birth continued to remain early (Perelli-Harris, 2005) and the postponement of first births was less pronounced in Ukraine than in other low-fertility countries (Aksyonova, 2022). Instead, Ukrainian women postponed second births (Perelli-Harris, 2008; Hilevych & Rusterholz, 2018), potentially due to material uncertainties, lack of housing, and insufficient family support, (Hilevych, 2016). In the post-Soviet period, researchers speculated whether low fertility stemmed mainly from ideational change, encapsulated by the Second Demographic Transition, or social anomie and the overall economic uncertainty which emerged after the collapse of the Soviet Union (Sobotka, 2011). Pronatalist policies like cash transfers for childbirth were seen as relatively ineffective for increasing or maintaining fertility (Frejka et al., 2016). Environmental factors may also have limited progression to second birth (Wesolowski, 2015). However, the effects of various uncertainties on fertility—both economic and those linked to war and displacement—have not been studied empirically in post-2014 Ukraine.

## Methodology

In this study, we used focus group methodology to explore the reasons underlying low fertility in Ukraine. Focus groups elicit social perspectives essential for understanding context-specific phenomenon and generating hypotheses (Morgan, 2018). Unlike in-depth interviews, which tend to emphasize biographical events, focus groups allow for a variety of responses and opinions, which often emerge during participants’ interaction. When answering questions, participants inevitably draw on their own, family members, and friends’ experiences, but they also reflect general observations about the social world around them. Hence, focus groups reveal shared public perspectives, but also the diversity of opinion.

We engaged the Kyiv International Institute of Sociology (KIIS), a Ukrainian research agency, to assist with recruitment and moderation. We conducted four groups in four types of locations with diverse profiles in the eastern part of the country, which had been most affected by the conflict: Mariupol, a medium-sized coastal city which had received thousands of internally displaced persons; rural areas not far from the separatist territories; Kharkiv, a large, fairly prosperous city in the northeast; and Donetsk, the capital city of the oblast, controlled by Russia-backed secessionists since 2014. Although we had planned to conduct the groups in person in summer 2020, the COVID pandemic made travel impossible, so we used Zoom. This approach had the advantage of providing access to participants living in the NGCA, where few foreigners or researchers were able to travel, even before COVID.

KIIS recruited participants in local public areas from a database of prior survey participants and with snowball methods. A preliminary screening interview conducted via Computer Assisted Telephone Interviewing ensured participants met age (18–45 for all groups), gender, and IDP status criteria. Within each site, we stratified the four groups by gender and IDP status. Grouping respondents by gender, and ensuring moderators’ gender matched that of the group, helped participants feel comfortable speaking on potentially sensitive topics relating to family and fertility (Axinn & Pearce, 2006). Because IDPs are often difficult to find in the general population, we targeted those who had moved after 2014 and tailored some questions to their situation (not analysed in this study). In total, we conducted 16 focus groups, with around eight participants in each.

The focus group discussions were moderated by KIIS professionals who had prior experience interviewing internally displaced persons. The ethics protocol for the study was approved by the University of Southampton. Participants gave oral consent to participate, were free to exit the interview at any time, and received a small compensation. The discussions were conducted in Russian, which is more commonly spoken than Ukrainian in the eastern regions. The online format resulted in a relaxed atmosphere, as participants joined the discussion from the comfort of their own homes. Because participants were physically separated, the virtual set-up even appeared to be less intimidating, resulting in people freely expressing their opinions, with all participants contributing and often interacting with each other.

The lively discussions were guided by a set of pre-determined open-ended questions about fertility decline, depopulation, partnership formation, and childbearing intentions. They started with general questions about problems over the past year and experiences with either displacement or life during the conflict. We asked about living conditions in informants’ localities, with special questions in the IDP groups about experiences of displacement, without referring explicitly to political issues. This battery of questions provided a general sense of whether they thought low fertility and population decline in Ukraine was a problem. We then asked how, if at all, the conflict impacted family relationships and partnerships. The block of childbearing questions delved deeper into the fertility situation in Ukraine, asking participants whether they thought the number of children born was too few, too many, or just right. Participants were asked to reflect on factors influencing people’s decisions and constraints to childbearing. They also discussed whether there was a stigma about childlessness, having only one child, and single motherhood.

We used a variety of strategies to analyse the focus groups and answer the research questions. Each co-author watched the discussions online and read through the transcripts. The third author coded the transcripts in Atlas.ti using a thematic approach that identified and aggregated passages pertaining to themes such as ‘fertility decline/financial hardship’ or ‘fertility decline/little state support’. We then discussed and agreed upon the major themes about childbearing and fertility decline which structured the results section. Although we never explicitly used the term in a question, various aspects of the theme ‘uncertainty’ emerged organically, (“bottom up”) as we show below. Uncertainty as a concept was also of theoretical interest, as it is a pervasive theme in the fertility literature. Information on the composition of each group is provided in Appendix Table 1; however, the limitations of focus group methodology preclude systematic analysis of differences by gender, locality, migration status, marital status, number of children, or other characteristics.

## Results

### The Context of Uncertainty

We first asked about the largest problem facing participants in the past year. The discussions revealed how underlying uncertainty coursed throughout everyday life, reflecting external strain and causing personal stress. Many of the concerns would have been familiar in other parts of Europe in July 2021, a year after COVID lockdowns and economic recession. Some participants mentioned difficulties with their children’s online schooling, accessing medical care, and isolation. Others complained about rapid inflation, the cost of utility payments, insufficient wages, and job loss. Thus, economic anxiety was palpable as many participants felt they had slipped into poverty.

The armed conflict of 2014, however, was a unique source of lingering uncertainty, still present seven years later. Those who had been displaced still recalled the sense of trauma and shock when they suddenly had to leave home, as well as the challenge of adapting to a new location, even if now their lives felt more settled:

P5: You know, of course, [the shock] is not that sharp anymore. At first, of course, it was hard, mentally difficult somehow. It felt very unpleasant; well, not comfortable, to put it mildly. That is, you were just living your life, made some plans, built – and bang! Everything changed. Everything changed whether you wanted it to or not. And again, really the biggest fear was for our relatives. Because back then it was... Well, it was really a matter of life and death in 2014-2015. That is, I think everyone will support me and understand that it was very scary. Either you survive or you don't. I had to leave, because [my life] could have ended.

P2 Living in one city, then abruptly abandoning everything is a serious change.

P5: Yes, it's very hard.

P2 Losing a business, losing everything, and starting from scratch is not realistic.

P6 Yes, and going to bed with your documents in your hands.

P2: Yes. (Group 7, Village IDP women)

The recollection of “going to bed with your documents in your hands” aptly captured a state of intense uncertainty about having to flee upon awakening. A male IDP living in Mariupol noted he still woke up late at night as a tangible sign of his anxiety, despite feeling much safer now:

P7: Well, after the move, when we moved to Mariupol, here it became much safer… There are still a few... Well, for example, I still have the habit of waking up around 3 a.m. It's just that in the moments when I lived [near the shelling], I woke up from every rustle, because I was very afraid. It's pretty calm now, but there's still a remnant left. (Group 1, Mariupol IDP men)

Although by July 2021 Mariupol had experienced six years of relative calm, another IDP in the Mariupol male group recounted how he had fled there with his wife to escape the fighting in Donbas only to experience a major rocket attack in January 2015, which left them rattled and inspired them to keep emergency suitcases packed in case they had to flee again:

P4: In Mariupol, of course, it's calmer. For some time, I was worried about all the shelling, which is somewhere in the distance, in the area of Shyrokyne or wherever it is taking place, exercises or whatever. But after the events in the East, each time for a long time, about a year or two, we lived in constant tension. Because as soon as things start to go “bang” somewhere, already our emergency suitcases were packed. Run, escape, and all that. Because no one knew what would happen and what would happen in the future. Well, it's calm for now, and I’m thankful for that. (Group 1, Mariupol IDP men).

Even long-time residents of Kharkiv, which experienced tension in 2014–2015 but avoided fighting and attacks, spoke of how the armed conflict had affected them psychologically and economically:

P5: Well, let's just say [the conflict] affected me quite a lot. That is, first of all, it had a psychological impact at first. I mean, for me this whole situation...although it didn't affect me personally, I still took it hard. I had depression for several months because of it. Then, a couple of years later, due to the deterioration of the economic and political situation in Ukraine, the branch of the company where I worked was closed. That's why I switched to freelancing. That is, quite a big change in life. And in general, my life has become less stable. (Group 10, Kharkiv local men)

These discussions illustrate how the conflict had negative repercussions even for those who were not forced to flee and did not experience direct attacks, including economic consequences and poor mental well-being.

### Explanations for Fertility Decline: Economic Constraints

Asked why fertility in Ukraine was low, many participants cited financial explanations: salaries were too low, costs of living were too high, and people were struggling to feed and clothe themselves, making multiple children simply unaffordable. As a result, having one child was hard, two more of a struggle, and more than two generally out of the question. Participants often explained their decision to have only one child as driven by financial concerns:

“Moderator (M): tell me, does that mean that most residents of Ukraine, of Kharkiv, have just one child?

P2: I think it is connected with finances. [Parents] can provide everything for one child, they are confident of that. That is, whatever the child needs. But if there are four kids – that will be too much to afford, materially. People understand that, so they simply don’t have [more than one].” (Group 10 Kharkiv local men)

The main priority is to invest in the one child that you already have, providing food, clothes, and education, which is the reason why most have only one child, or possibly two.

P1: Our acquaintances, all of them, have two kids at the most, and the bulk of them have one. I have one child. And as most people probably think now, I want to give him the best of everything: [for him] to eat well, dress well, look good, attend different places and after school activities. (Group 2, Mariupol local men)

Participants often gave the impression that having a first child is not a decision, but a naturally occurring event expected to happen. But the question of additional children after the first was a matter to be decided rationally, with economic factors featuring prominently in the criteria for making that choice:

P7: Well, that's right. The first child is love, and the second child is an apartment, a salary.

M: Okay. Thank you. I'm wondering: P7 is saying that the first one is more of that kind of relationship, and the second one is already a rational...

P7: Yeah, yeah. It is like when you've already seen what it's like with the first one. And you already have an apartment and job, and then you think you can have the second one and the third one. (Group 13, Donetsk men)

Many voiced very precise financial complaints, for example, increasing utility payments. Their reference to the specific salaries needed per child and exact knowledge of prices of products indicated a mental calculation of how many children they could afford:

“P2: People are not ready to have 3-4 children, they don’t want to because they cannot afford it materially. A husband who earns 40,000 [hryvnia], a wife who works somewhere, I don’t know, at home, gets 10. That’s enough for two kids. So, they don’t want 3-4. Not because they don’t love children, but simply because they won’t have the finances for when those kids are 8-10 years old. They have to buy everything, clothes, shoes. It’s just very difficult to raise kids, very hard to support them.” (Group 10, Kharkiv local men)

In general, more than one child, especially three children, were seen as an “excess”, “luxury”, or “expensive pleasure” (Group 7, Village IDP women). Some also disapproved of very large families, criticizing people who had more children than they could afford:

P4: I don’t want to sound rude, but let’s say that people who think a little, when it comes to having children, they think first of all whether they are able to provide for them. But some people, well, they reproduce like cockroaches hiding under a fence somewhere. And they give birth to 10 children who run around in hand-me-downs. Can it be that the demographic crisis in Ukraine will be solved that way? Once again – what will become of those children who are ignored by their parents, who cannot provide for them, educate them, clothe them, and so on? (Group 1, Mariupol IDP men)

Despite the overall despair expressed about the poor state of the economy, a few participants were optimistic about their financial situation and their plans for children. For example, a woman with one child in occupied Donetsk said she was looking forward to her second child, and that many in her network had two children.

P4: It is some kind of “boom.” Everyone I know has started having their second child, even their third. I don't know what it is connected to (Group 16, Donetsk women).

Some participants in Mariupol also felt that their cities were experiencing a recuperation, even a baby boom. This increase in mothers with baby carriages was contrasted with the period in 2014–2015, when the threat of war was more acute (Perelli-Harris & Hilevych, 2023).

Despite some variation in childbearing intentions and plans across focus groups, the participants often expressed the view that low fertility in Ukraine was a symptom of their poor economic situation. In contrast to Italy and Spain (e.g. Gatta et al., 2022; Lebano & Jamieson, 2020), Ukrainians’ economic concerns were not primarily about job instability or temporary contracts, but instead about low wages and the inability to afford essentials. Some of our participants with children disclosed they were struggling to feed and clothe them. Thus, our participants were concerned about basic survival. Undoubtedly, these economic concerns were prevalent and directly influenced those who still did not have as many children as they would like.

### Generalized Uncertainty and Fertility

Underlying the discussions about economic constraints was a pervasive sense of uncertainty that was sometimes difficult to define. The participants themselves did not always point to the source of uncertainty and sometimes spoke of instability in vague terms. Sometimes it was evident that the military incursion and resulting displacement were responsible; at other times, respondents clearly referred to economic woes. But without a doubt, uncertainty generated a pessimistic outlook that led to an inability to plan one’s future, including having more than one child. This man in Mariupol stated bluntly how instability led to low fertility.

P2: Economic problems, uncertainty about tomorrow, there is no stability. People do not believe that tomorrow will be better. That is why they do not give birth. (Group 2 Mariupol local men)

When discussing the reasons for low fertility, these women from rural Donetsk talked about the inability to afford additional children and meagre child benefits, but also a lack of stability in life, which was not considered a problem for previous generations.

P7: Families used to have two or three children. But now, if young people have one child, they either can't afford more or they decide not to because of the current situation.

M: How is that reflected?

P8: Also, because of the [low] payments for children [from the government]

M: How is that reflected?

P2: There is no stability in life. (Group 8, Village local women)

Most of the narratives about uncertainty did not elucidate the sources of the uncertainty and were not specific to individuals’ own decision-making processes. Instead, they expressed emotions about the situation in Ukraine and perceptions of low fertility more generally. For example, this discussion was in response to a question about why Ukraine’s overall population is declining:

P8: I think it's just kind of fear and uncertainty about the future. Not everyone has a job, their own place to live, and it's also kind of going to be a lot harder once you have a child. I think because of that.

P1: That too, yes, I agree. That too, yes.

M: P5, would you like add anything?

P5: Basically, all the speakers have already said everything. Uncertainty about tomorrow, I think, is the main issue. (Group 9, Kharkiv IDP men)

One woman from Donetsk said that people’s decisions to have children were personal, with each having their own reasons. At the same time, she expressed feelings of anxiety and dread for the future, especially for one’s children.

P3: In the old days it was mandatory [to have children]. And now it's everybody's own personal business, let’s just say. Moreover, you understand, people are now afraid, the future is unclear. And you think with dread, what's in store for your child, what's going to happen in general. It's just scary, you read all those prognosticators, psychics, politicians, they are forecasting everything. And sometimes with horror you think about what will happen next and what kind of future will your child have? (Group 16, Donetsk women)

The quote above also revealed how participants commonly heard forecasts about a bleak future, particularly through social media. Some alluded to conspiracy theories: an unknown force had created a fake war, or a fake virus, which influenced people to not have children. Some mentioned pollutants and toxins, 5G, or Bill Gates, as responsible for increasing infertility, not only in Ukraine, but around the world.

P4: That is how everything goes, and how everything has always been. ‘People die, [there is] war. Then, there is the virus, then there are many other factors that motivate one not to give birth. Because it is hard to sustain a child. … You can try to have one, but two-three, no comments. Everything is moving in that direction. Why is this all taking place? Who is negatively affected by this? And then…we all are, in my view, like cockroaches. Those [people] who are behind all of this …those who are doing this, they are doing this on purpose. Science has proved long ago that overpopulation is happening on planet earth. And then, in some way, the question is… we are not being asked, unfortunately, whether we want to live or not, [they] mow us all down, everyone in a row.

M: And who does it all?

R4: I don’t know who does it

R3: Americans (smiling). (Group 7, Village IDP women)

Participants also directly blamed the government for their personal situations. One man from the rural Donetsk region discussed how population decline is connected to lack of certainty in the future and how the government prolonged this uncertainty by raising taxes, utility payments, and low child payments:

P2: But here it has to do mostly not with the conflict as such, but with uncertainty about the future. The state is doing everything so that we do not feel certain [in the future]. Taxes are being raised, utilities are being raised. But for some reason, the wages increase very slowly. People do not keep up with it all. That is why people are concerned, what if tomorrow they [prices] will rise even further, then how will they feed their children? (Group 6, Village local men)

These discussions reflect how generalized uncertainty about finances and the future is linked to a lack of trust in the government, the economy, and society in general. The conspiracy theories were especially indicative of anxieties about what will come next. Many of the discussions revealed how Ukrainians often felt a loss of agency and control over their own lives. This lack of confidence in the future prospects is linked to why some would curtail childbearing.

### Uncertainties of Armed Conflict and Displacement

The feeling of uncertainty was reinforced by discussions about the frozen conflict between the Ukrainian government and Russian-backed separatists. The fighting had produced extreme psychological strain, especially among IDPs, which made informants wary of having children or likely to postpone indefinitely. This woman’s observations about the number of children in Mariupol reflects local fertility trends, contrasting the period of stability with the period of fear and anxiety when fewer children were born.

P5: I work in a kindergarten, and I know that there was a very colossal shortage of children in the 1990s and early 2000s. Then there was a surge of children. Around 2005, or 2005-2006, there were more and more [children born]. There was a lot of help and support from the state. People were giving birth more eagerly and in larger numbers. There was stability. When the hostilities began, people tensed up a little bit. Because you don’t know, even if you live in Mariupol, maybe you will have to move somewhere else. We were afraid of that for a long time. That is, we constantly kept the gas tank of our car full so that in case [it became necessary] we could leave. And that's why I observe now that not so many children are born now. (Group 3, Mariupol IDP women)

Although hostilities at the time of the focus groups were less acute, people still recognised that the frozen conflict was ongoing and not a suitable environment to have children.

P4: This is probably an indicator of the state of the country in general, of stability. Because people who are well-balanced take this step in a balanced way, a child. They're thinking, “What's next?” How can you have a child in a country that is at war, where there is no stability at all? (Group 11, Kharkiv IDP women.)

In the NGCA, physical security was still a very live issue:

P1: Some people live in the center [of town], where you see a lot of people. Some live in the outskirts, where you still hear shooting or, god forbid, even witness fighting. Of course, they won’t be having any kids there (Group 14, Donetsk men)

IDPs, many of whom were still bitter about having to flee, discussed how the uncertainty affected their childbearing plans. While some said they had managed to find work and housing, others complained about how their situation remained fraught seven years later, after multiple moves, particularly with respect to finding suitable housing to rent, not to mention buying an apartment. Many IDPs in the two cities said they were undertaking programmes of study or had recently completed them, but could not find work. These participants in particular expressed fears for the future of their country:

P5. For me, let’s just say the situation in the country makes me worried. I generally don’t know what to expect in the near future. Maybe I will have to move once again. And if I have to flee again, it is harder with children – if I have to try to get set up in another country or in another place, it is not at all simple. (Group 10, Kharkiv local men)

A history of displacement also affected how the participants framed decisions to have only one child. One child may be necessary, but experience with instability can continue to influence the decision to have more than one.

M: Speaking of IDPs, are there any additional factors that are holding you back [about having children]?

P5: Of course, there are. I was talking to some people I know. They have one child. I said, "It is good timing that the old one is 7 years old, it's not too late to have a second." But no, it [displacement] influences, influences a lot. Instability always affects childbearing. As difficult as it is, people always want to have one child. And then they consider stability, the state of their family. The opportunities, rather, of the family. (Group 3, Mariupol IDP women)

Others pointed to examples of how the successive crises of the last years had led some couples to postpone even their first child indefinitely, especially in the NGCA and conflict areas. This man from Donetsk described how the war and ensuing economic instability can interact, leading to a lack of future prospects.

P1: We also have acquaintances with whom we go to the playground – they have been dating for five years, but they don’t plan to have children because of the pandemic and the conflict. Their home where they were living was bombed, so they now live in a rented apartment, they have no permanent work… They want kids, but they have no hopes for the future. (Group 13, Donetsk men)

Some who had directly witnessed armed conflict in the east and had either left their homes or endured the instability of staying in place were more anxious about the prospect of having children.

P4: With all the financial instability and danger, how can you have kids? How? In our situation to worry about kids… And how will things be for them in the future? You already start to wonder, what should you do – raise a kid here or gather up all the “Talmuds” and go somewhere else, where there are rights and some kind of future. You do not think about yourself only anymore, but about children and their future. (Group 13, Donetsk men)

Although most participants in our groups had only negative views of the military conflict, some found a silver lining, or at least emphasized how they and others adapted to challenges and found new opportunities. One IDP described first meeting her current husband as she fled her home in the Donbas. A male IDP in Mariupol said that by forcing him to leave his childhood home and family, the military conflict had spurred him to take control of his own destiny, make independent decisions, and find his path in life on his own. This included a decision to have a child:

P8. At the moment, the birth of my baby is the best thing that has happened in my life. Because before that, it was just a drudge. You live for yourself, and just live. And everything now is just bright colors, daughter, wife, walks.

M. Did the conflict have any effect?

P8. Yes, the conflict influenced the moment, because it made me “ripe” for action. Specific actions of an adult. If it weren't for [the conflict], there wouldn't be these other things. (Group 1, Mariupol IDP men)

Thus, having a child in the midst of conflict can bring about mixed reactions—on the one hand positive, because having children is a joy and a sign of adulthood, but on the other hand, an underlying worry. A man in one of the NGCA groups noted that he was deterred from having kids for financial reasons before the war, but now thinks that waiting for the “right moment” is a mistake, despite the ongoing shooting:

P2: That’s just what I wanted to say: we thought so too, that the time was not favorable for [having kids]. That is, we thought -- when we were in Ukraine, when everything was normal here, everything was fine, and we thought: no, we don’t have money, no, it is not yet the time to have children. And after that, they often had children in the DNR [Donetsk People’s Republic], when there was still shelling. So that's how it is. (Group 14, Donetsk men)

This man near the border of the separatist territories spoke of how a child came along in the midst of the conflict. While he said that the situation is stable now, his reference to having a child who will be a future “defender” of the motherland implies an expectation of future war.

R6: Well, I can speak for myself. We, you can say, started our family during the conflict. From the very beginning. And the baby was born in 2015.

M: So was it the conflict that affected it in some way, or was it...

R6: Turns out it had a positive impact? (chuckled).

M: What was the impact of the conflict? Tell me.

R6: Well, we were also afraid that we might have to leave [our village]. But nonetheless, things calmed down a little, and we stayed in the same place of residence. … we began to multiply, a child appeared. Now things are more or less stable, we are staying here. So, it is like we are raising a defender of the [Ukrainian] motherland. (Group 6, Village local men)

Thus, despite participants continuing to have children during the conflict, on the whole the discussions suggested that societal-level crises led to individual level perceptions of instability and produced a lack of confidence in the future. Many of the discussions revealed an eerie prescience about the massive war to come:

P1. Maybe people even want to [have children], but there’s the conflict, and it’s uncertain what will happen next. There will be more pandemic, there will be more war, there will some other kind of conflict – there is no stability, no confidence in what tomorrow will bring, so [they] don’t have kids. (Group 13, Donetsk men)

## Conclusions

Over the past decade, Ukraine has experienced a series of major crises, including economic recession, political turmoil, and pandemic, crises which are not unique to Ukraine. The experience of armed conflict, however, has added a distinct dimension of existential uncertainty. Research in African countries has linked existential uncertainty to high mortality rates due to the HIV pandemic (Trinitapoli & Yeatman, 2011). In eastern Ukraine, even before Russia’s full-scale invasion in 2022, existential uncertainty was rooted in the experiences of war and displacement. Many of our participants directly witnessed shelling and fighting during the war in Donbas, leading to a visceral fear for survival and awareness of the fragility of life. For others, even the knowledge of nearby combat produced a sense of foreboding. People who had been displaced still slept with documents in their hands, kept their gas tanks full, maintained packed emergency suitcases—small but telling signs of genuine fear that they could have to flee yet again. These disparate sources of uncertainty interacted: the threat of armed conflict led to psychological strain; displacement added to many people’s material woes; and unemployment or insufficient wages resulted in a struggle to meet basic needs. This uncertainty resulted in a wariness of governmental authorities and lack of confidence in the future. Our focus group participants themselves saw instability as a primary reason why fertility in Ukraine was so low.

Uncertainty is an abstract concept. In some cases, those experiencing it may not perceive its precise source. Yet, concrete economic and political developments, by themselves and in combination, trickle down into general social unease and apprehension about the future, which then colours couples’ decisions to have children. These discussions reflect what Vignoli et al (2020) call “narratives of the future”, or “imagined futures embedded in social elements”. Potential parents naturally imagine what life will be like for themselves, and whether the future will be suitable for their children. In our focus groups, many individuals projected their feelings of uncertainty into the future—anticipating another war, pandemic, or further economic deterioration—and were pessimistic about childbearing. Nonetheless, some participants responded to these factors by “seizing the moment” to get married and/or have children. They admitted that these adverse experiences challenged them to reevaluate their lives and invest in children. Some were even relatively optimistic about the future, particularly since the conflict in the Donbas had been “frozen” for 6 years at the time of the focus groups. Thus, although the war had brought considerable disruption, some participants had reoriented themselves to their new lives.

Our research provided evidence on how macro-level uncertainty trickles down to shape how people think about having children in Ukraine by highlighting two different dimensions of uncertainty. Discussions about generalized uncertainty usually emerged when we asked opinions on the reasons for Ukraine’s population decline. The discourses about instability often referred to “people”, “couples”, and social norms or attitudes, not their own decisions or attitudes. However, economic uncertainty was the primary reason curtailing individuals’ own childbearing. Financial concerns were pervasive and often precise, suggesting that couples mentally calculated a cost–benefit analysis about how many children they could afford. The discussions reflect Becker’s thesis in which couples consider trade-offs between the “quantity” and “quality” of children (Becker, 1960). Ukraine’s population is relatively well-educated and can control fertility through contraception or abortion, which the participants agreed were still widely available during the armed conflict and pandemic. Taken together, this is a recipe for extremely low fertility.

Nonetheless, most focus group participants expressed a basic desire to have at least one child, in accordance with previous studies of Ukraine (Hilevych, 2020; Perelli-Harris, 2008), but in contrast to findings from very low-fertility countries in Southern Europe where people indefinitely postpone first births due to ambivalence (Lebano & Jamieson, 2020). Many of our participants wanted to have a first child, even if conditions were imperfect. Because having a first child was largely automatic once couples married, the main decision-making process and concomitant economic uncertainty emerged when considering subsequent births. Having a second child was seen as a more rational choice that depended on factors like having an apartment, a decent income, and stable material conditions necessary to “stand on one’s feet”. Some of our participants and their friends had achieved this stability and had had or were planning a second child, even in separatist-occupied Donetsk. For others, however, a concerted decision to have a second child did not seem to be in the cards, as the conditions were unlikely to ever be quite right. In such cases, perpetual uncertainty and ambiguity may lead to indefinite postponement. As in the African context, Ukrainians may have maintained an ideal number of children, but the actual number of children born was more flexible and a “strategic response to life’s circumstances” (Trinitapoli & Yeatman, 2018).

The discussions also revealed how misinformation, distrust of authorities, and conspiracy theories can shape attitudes towards fertility. Such factors were especially salient in our informants’ explanations of Ukraine’s fertility decline. Participants expressed, if often implicitly, a lack of agency over their own lives, as if an outside power was in control. Without social trust and cohesion, societies do not have the sense of security that can buffer the negative impact of uncertainty on fertility (Aassve et al., 2020).

Our theoretically motivated aim has been to explore how economic and political sources of uncertainty shape considerations about childbearing in Ukraine. Other common themes in the low fertility literature, such as opportunity costs or gender equity, only came up a handful of times across the sixteen groups. Additional factors did arise, but we lack space to discuss all of the reasons provided for low fertility. For example, some participants stressed the requirement to have a high-quality relationship before having children; high levels of divorce and separation in Ukraine have meant that some couples have not had the stable relationships necessary for a second child. Others mentioned the lack of childcare availability, either from the state or their own parents who may have been far away. COVID19-related shutdowns and alcohol consumption were also cited as potential influences, as both potentially encouraging and deterring childbearing. All of these considerations led into other facets of life uncertainty, which can reduce fertility.

Now, as Russia’s assault on Ukraine continues, it is impossible to accurately measure or predict Ukrainian fertility rates. Nonetheless, our 2021 focus groups reveal three points to consider about the future of Ukraine’s fertility. First, the instability caused by the 2014 separatist conflict likely reversed an uptick underway since the early 2000s, returning fertility to very low levels. In the views of many informants, young people and those directly affected, including IDPs and NGCA residents, postponed childbearing. The resulting decline in TFRs could be a tempo effect, but short-term postponement can become long-term or even indefinite postponement, eventually resulting in lower completed fertility. Second, the armed conflict resulted in economic uncertainty and a lower standard of living, which eroded confidence in the future. Third, despite these negative effects, the desire to have at least one child generally remained strong, and the main decision about childbearing was around two or three children. These results suggest that while fertility will largely be postponed during the first years of the war, leading to world-record lows, Ukraine is likely to experience a minor baby boom as postponement ends and people regain sufficient confidence in the future to have children.

In conclusion, this study elucidates how crises produce individual-level uncertainty which may then translate into fertility intentions. Our focus groups revealed multiple dimensions of uncertainty, both on the individual and societal level, that permeate cognitive processes around childbearing decision-making. In particular, the experience of armed conflict and displacement in Ukraine led to existential uncertainty unlike any other low-fertility country in the world. Given the prevalence of wars throughout the world—not least in Ukraine itself –further theoretical attention and empirical research are needed to better understand how armed conflict produces cascading, overlapping uncertainties and how these impact fertility decisions.

## Data Availability

These data cannot be made publicly available due to the sensitive nature of the material. Although the transcripts have been anonymized, the participants may have disclosed personal information and opinions, which could potentially be used to identify them. Given some of the respondents may now be living in Russian-occupied territory, this information could be used against them in a harmful manner.

## Appendix

See Table 1.

**Table 1:** Composition of virtual focus groups

Group	Locations	Sex	IDPs?	Marital Status				Number of kids			
				Married (%)	Cohabiting (%)	Was married (%)	Never married (%)	0 (%)	1 (%)	2 (%)	3 (%)
1	Mariupol	M	IDP	50	13	13	25	50	50	0	0
2	Mariupol	M	Local	60	10	0	30	60	30	0	10
3	Mariupol	F	IDP	50	0	0	50	38	50	13	0
4	Mariupol	F	Local	25	25	38	13	50	50	0	0
5	Villages (Donetsk)	M	IDP	25	13	25	38	50	13	25	13
6	Villages (Donetsk)	M	Local	50	25	0	25	50	50	0	0
7	Villages (Donetsk)	F	IDP	50	0	25	25	25	50	25	0
8	Villages (Donetsk)	F	Local	50	13	0	38	38	25	38	0
9	Kharkiv	M	IDP	38	13	13	38	50	38	13	0
10	Kharkiv	M	Local	38	25	25	13	38	50	13	0
11	Kharkiv	F	IDP	25	38	25	13	25	38	38	0
12	Kharkiv	F	Local	50	13	0	38	38	25	38	0
13	Donetsk city	M	Local	50	25	0	25	13	63	13	13
14	Donetsk city	M	Local	25	13	13	50	50	38	13	0
15	Donetsk city	F	Local	63	0	25	13	13	50	38	0
16	Donetsk city	F	Local	38	13	38	13	38	38	25	0

Group	Locations	Sex	Age			Employment Status				% University graduates
			Min	Max	Average	Working (%)	Unemployed (%)	Student (%)	Other (%)	
1	Mariupol	M	18	45	32	75	0	25	0	63
2	Mariupol	M	18	45	30	90	0	10	0	40
3	Mariupol	F	24	44	34	88	0	0	13	75
4	Mariupol	F	18	45	33	88	0	13	0	38
5	Villages (Donetsk)	M	19	45	32	63	13	25	0	25
6	Villages (Donetsk)	M	20	42	29	63	13	25	0	50
7	Villages (Donetsk)	F	24	45	33	63	25	0	13	50
8	Villages (Donetsk)	F	18	45	31	88	0	13	0	25
9	Kharkiv	M	18	45	31	75	13	13	0	88
10	Kharkiv	M	19	40	29	88	13	0	0	75
11	Kharkiv	F	27	45	35	88	13	0	0	63
12	Kharkiv	F	18	45	33	88	0	13	0	75
13	Donetsk city	M	19	45	35	38	38	13	13	38
14	Donetsk city	M	23	43	30	88	13	0	0	63
15	Donetsk city	F	19	45	35	63	0	0	38	50
16	Donetsk city	F	29	45	37	63	38	0	0	63

Group 2 had 10 informants; all others 8. Donetsk city was non-government controlled area (NGCA)

## Footnotes

1 Surveys on fertility, such as the 2007 Demographic Health Survey and the Ukrainian Longitudinal Monitoring Survey (last conducted in 2012), pre-date the Russian-backed separatist uprising and thus are unsuitable for exploring the impact of armed conflict.
